# Iron Compounds in Anaerobic Degradation of Petroleum Hydrocarbons: A Review

**DOI:** 10.3390/microorganisms10112142

**Published:** 2022-10-29

**Authors:** Ana R. Castro, Gilberto Martins, Andreia F. Salvador, Ana J. Cavaleiro

**Affiliations:** 1 CEB—Centre of Biological Engineering, University of Minho, 4710-057 Braga, Portugal; 2 LABBELS—Associate Laboratory, 4704-553 Braga/Guimarães, Portugal

**Keywords:** petroleum, anaerobic, biodegradation, iron oxides, BTEX, PAH, alkanes, direct interspecies electron transfer, ZVI

## Abstract

Waste and wastewater containing hydrocarbons are produced worldwide by various oil-based industries, whose activities also contribute to the occurrence of oil spills throughout the globe, causing severe environmental contamination. Anaerobic microorganisms with the ability to biodegrade petroleum hydrocarbons are important in the treatment of contaminated matrices, both in situ in deep subsurfaces, or ex situ in bioreactors. In the latter, part of the energetic value of these compounds can be recovered in the form of biogas. Anaerobic degradation of petroleum hydrocarbons can be improved by various iron compounds, but different iron species exert distinct effects. For example, Fe(III) can be used as an electron acceptor in microbial hydrocarbon degradation, zero-valent iron can donate electrons for enhanced methanogenesis, and conductive iron oxides may facilitate electron transfers in methanogenic processes. Iron compounds can also act as hydrocarbon adsorbents, or be involved in secondary abiotic reactions, overall promoting hydrocarbon biodegradation. These multiple roles of iron are comprehensively reviewed in this paper and linked to key functional microorganisms involved in these processes, to the underlying mechanisms, and to the main influential factors. Recent research progress, future perspectives, and remaining challenges on the application of iron-assisted anaerobic hydrocarbon degradation are highlighted.

## 1. Introduction

Petroleum-derived oils are still the most important primary energy source in our society and represent an important fraction of the economic markets. Additionally, petroleum is a key raw material for a wide range of non-fuel products, such as solvents, lubricants, and other compounds that are, in turn, used as raw materials in the petrochemical industry (e.g., naphtha, ethane, propane, ethylene, propylene) [1].

In the last decades, the activities of the oil and gas (O&G) industry have contributed to the occurrence of oil spills throughout the world, both on land and in marine environments, with dramatic consequences to the ecosystems and ultimately to human health [2]. Releases of petroleum can occur during routine operations of extraction, production, transportation, refining, and storage processes, and also during illegal disposal practices such as direct wastewater discharge [3,4,5,6]. Given that the O&G industry is prevalent worldwide, and the rate of oil consumption is predicted to increase, minimization of the environmental hazard of its activities will continue to be a challenge [7,8].

Besides oil spills in the environment, considerable amounts of different hydrocarbon-contaminated wastes and wastewaters are produced by several types of oil-based industries. Wastewaters generated during oil production, as well as oily sludge, are considered as the most relevant [9]. The handling and discharge of oily waste and wastewater has been progressively regulated and today the O&G sector needs to cope with tight restrictions. Due to the complex structure, toxicity, and harmful effects of these wastes, an effective treatment is imperative [10].

The use of anaerobic microorganisms, able to consume petroleum hydrocarbons under oxygen-limited conditions, can be a suitable solution to treat both environmental contaminated matrices and oily waste/wastewater. Anaerobic degradation processes can take place in situ, in deep contaminated environments, such as soils, sediments, and aquifers [11,12,13], or can be performed ex situ using different types of bioreactors [14,15]. In this case, part of the energetic value of these compounds can be recovered in the form of biogas, which can be upgraded to biomethane and injected into the natural gas grid, thus contributing to clean and more resilient energy systems.

**Figure 1:**
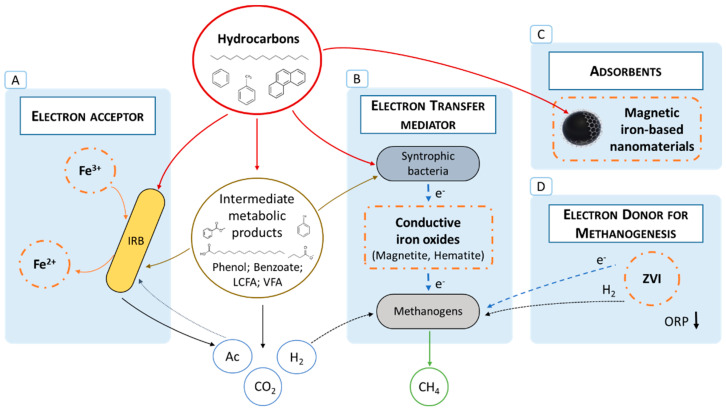
Scheme of the potential roles of different iron compounds in anaerobic degradation of petroleum hydrocarbons: Fe(III) as electron acceptor (**A**); conductive iron oxides as electron transfer mediators in hydrocarbon conversion to methane (**B**); magnetic iron compounds as hydrocarbon adsorbents (**C**); and zero-valent iron (ZVI) as electron donor for methanogenesis (**D**). IRB—iron-reducing bacteria; LCFA—long chain fatty acids; VFA—volatile fatty acids; Ac—acetate; ORP—oxidation-reduction potential.

The positive effects of iron compounds in anaerobic digestion (AD) processes were recently reviewed by Tian and Yu [16] and Li and colleagues [17], mainly reporting iron-assisted AD with some simple substrates (glucose, acetate, propionate, butyrate) and a few complex wastes (waste-activated sludge, food waste, rice straw, and swine wastewater) [16], but only scarcely addressing hydrocarbons. Iron compounds may play multiple roles in the removal and degradation of petroleum hydrocarbons, thus contributing to enhanced biodegradation and bioremediation (Figure 1). Its multiple roles are discussed in detail in Section 5, Section 6 and Section 7. Besides its potential effect as an electron acceptor in microbial hydrocarbon degradation (Figure 1A), iron is also an essential element of different enzymes and co-factors. Depending on the iron species, it can also function as a buffer for organic acids or as an electron donor for enhanced methanogenesis (Figure 1D), thus promoting the conversion of hydrocarbons to methane. Conductive iron minerals have also been reported to stimulate electron transfers in methanogenic processes [18], although only few works addressed this effect in the context of petroleum hydrocarbon biodegradation (Figure 1B). Nevertheless, this topic was identified as a “Crystall Ball” feature that will drive innovative research in the field [19]. Iron compounds can also act as hydrocarbon adsorbents (Figure 1C). In sum, the application of iron compounds to anaerobic processes can improve the system’s performance, and potentially lead to increased financial returns of in situ and ex situ hydrocarbon bioremediation processes [20].

This review is focused on the multiple roles of iron compounds in petroleum hydrocarbon biodegradation under anaerobic conditions. The presence of petroleum hydrocarbons and iron compounds in industrial waste/wastewaters and in nature is reviewed. Current knowledge on key microorganisms and metabolic steps involved in the anaerobic biodegradation of hydrocarbons is summarized. The diverse effects of iron in these biological reactions are highlighted and reviewed, and the most important advances in the field are summarized, as well as their potential to boost hydrocarbon removal and anaerobic degradation.

## 2. Petroleum Hydrocarbon Types, Sources, and Occurrence in Waste/Wastewater

Petroleum hydrocarbons can be broadly divided into aliphatic (including linear and branched chain alkanes, as well as naphthenic compounds) or aromatic, e.g., BTEX—benzene, toluene, ethylbenzene, and xylene isomers (*o*-xylene, *m*-xylene, *p*-xylene)—or polycyclic aromatic hydrocarbons (PAHs) [21,22]. Several mono- and polyaromatic hydrocarbons are among the 30 compounds most frequently detected in groundwater, according to data from the UK Environment Agency’s monitoring program of organic pollutants [23]. In general, hydrocarbons are largely apolar and are chemically stable, presenting minor reactivity at room temperature [24]. Many PAHs are carcinogenic and/or mutagenic, and tend to bioaccumulate within organisms due to their hydrophobicity and low water solubility [25]. A total of 16 PAHs were included in a list of priority-control pollutants defined by the United States Environmental Protection Agency [26].

**Figure 2:**
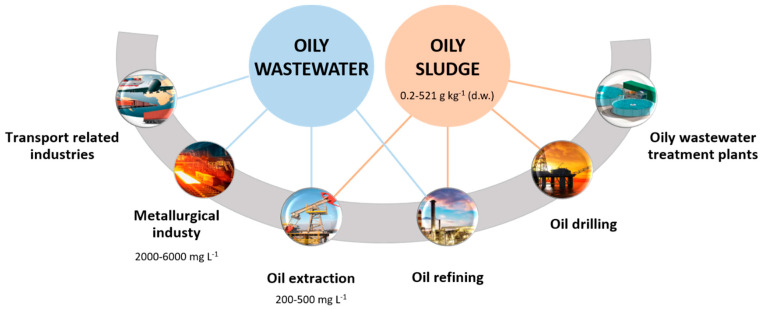
Different sources and average hydrocarbon concentrations found in oily wastewater and oily sludge.

Worldwide, hydrocarbon-contaminated wastewater production was estimated at 5.3 million m^3^ per day in 2009 [27], resulting from different sources (Figure 2), namely crude oil extraction and refinement, lubricants and petrochemical manufacturing, the metallurgical industry, transportation industries, automotive repair stations and industrial equipment maintenance [28,29]. During crude oil drilling and extraction, produced water (PW) is the main oily wastewater generated [30], and may reach up to 39.5 million m^3^ day^−1^ globally [31], although used fracking and drilling fluids, as well as wastewater from maintenance procedures, are also produced [29]. PW is an oily wastewater that consists of a saline aqueous solution containing hydrocarbons. PW results from the injection of large amounts of seawater in offshore reservoirs, to increase the oil recovery rate during the oil extraction process, and subsequent separation of aqueous and oily phases [31]. The worldwide production of PW is increasing concomitantly with the growth of O&G production, despite the fact that the majority is reutilized [32,33,34]. In PW, aliphatic hydrocarbons, BTEX, and PAHs are important pollutants (Table 1). The typical range of total petroleum hydrocarbon (TPH) concentration generally averages 200–500 mg L^−1^ [35,36,37], although TPH concentrations up to 7220 mg L^−1^ have also been reported [36].

Another relevant source of oily wastewaters (Figure 2) is the petroleum refining industry (crude oil transformation into several petrochemical products), particularly from the distillation units, cooling, desalting, and boiling systems, hydrotreating, cracking procedures, lubricants for machinery, spent caustic, and ballast water [28,38]. They are formed from the contact of water with crude oil products throughout the refining process, and contain oil and greases, phenols, suspended solids, cyanides, sulfur and nitrogen compounds, and different heavy metals, namely iron [39,40]. The chemical composition of these wastewaters, as well as their hydrocarbon content, varies depending on the processes applied in each refinery [39]. As an example, a petroleum refinery wastewater with 270 mg L^−1^ TPH was reported by Gargouri and his team [41]. Petroleum refinery wastewater generally has more PAHs and fewer lighter hydrocarbons than crude oil [39].

The metallurgical industry also originates considerable amounts of oily wastewater during metal manufacturing and processing (such as solvent extraction and electroplating). For example, in China, wastewater discharged from primary iron and steel plants reached 0.63 billion m^3^ in 2011 [42]. Metallurgical wastewaters generally contain emulsified oil, ranging from 2000–6000 mg L^−1^ up to 14,000 mg L^−1^, as well as suspended solids, emulsifiers, surfactants, degreasing agents, solvents, metals, and acids/alkalis [42].

The continuous increase in the activities of transport-related industries is contributing as well to the generation of hydrocarbon-containing wastewaters, particularly from automotive repair and wash stations and industrial equipment maintenance units [29].

Oily sludge is composed of oil, solids, and water, and is mainly generated during crude oil routine exploration processes, accumulating in the bottom of storage and transportation tanks and pipelines, and also in hydrocarbon-containing wastewater treatment plants (Figure 2). It includes mud from the drilling process, oily wastes from the wells and emulsified oils from the petroleum refining process [43]. Around 60 million tons of oily sludge are produced every year and more than one billion tons are accumulated around the globe [44]. TPHs account for 0.2 to 521 g kg^−1^ of sludge dry matter [45], with alkanes and aromatics representing 40–52% and 28–31%, respectively [46].

## 3. Biogeochemical Iron Cycle and Natural/Anthropogenic Sources of Iron Compounds

Iron is widespread all over the Earth’s crust and it is considered one of the most abundant elements on the planet. Iron redox cycling has an important role in the degradation and fate of organic contaminants, such as petroleum hydrocarbons, via oxidative and reductive transformation processes. These processes depend on iron speciation and dosage, accessibility, crystallinity, and microbial activity, as well as on the type of hydrocarbons or hydrocarbon mixtures [47,48,49,50].

In the environment, the different iron species have different physicochemical properties that determine the extent and inhibitory or stimulatory influence on microbial communities [50]. Table S1 presents the chemical formula, the crystal structure, and the appearance of the different iron species. Iron is present essentially in two main redox forms. One is ferric iron [Fe(III)], which is almost insoluble at neutral pH, and precipitates as Fe(III) minerals both in oxic and anoxic systems (Table S1) [47,51]. The other is ferrous iron [Fe(II)], which is mostly soluble at neutral pH and, therefore, more bioavailable. Formation of Fe(II) minerals may occur in anoxic environments, but not in the presence of oxygen, since O_2_ quickly oxides Fe(II) to Fe(III) [47,51]. Crystallinity critically affects the extent of the bioavailability of iron and refers to the degree of the 3D order of the atomic lattice [50]. For example, ferrihydrite and Fe(III) oxyhydroxide present short-range ordered structures, while hematite, magnetite, and goethite present a long-range ordered structure [52]. Another important property is the electric conductivity. The different iron species present different values of electric conductivity, ranging from the insulative (e.g., ferrihydrite), semiconductive (e.g., hematite), and conductive forms (e.g., iron (III) chloride) [50]. Electric conductivity could influence the way iron compounds interact with microorganisms, such as in the case of the enhancement of methane production by anaerobic microbial communities [18]. Regarding magnetism, this property could influence the electrostatic exchange between iron and the surrounding environment. More detailed discussions about the physicochemical properties of the different iron compounds (namely solubility, crystallinity, conductivity, and magnetism) were summarized by Baek and colleagues [50].

Besides the referenced chemical reactions, iron cycling is also influenced by the biological activity of different microorganisms. In fact, microbial-based iron transformations are faster than chemical transformations [52]. These processes can take place in several natural systems, such as aquatic environments, sediments, and soils, as well as in anthropogenic environments (e.g., anaerobic bioreactors). In anoxic and/or oxic environments, microorganisms are able to oxidize Fe(II) into Fe(III) and reduce Fe(III) to Fe(II). The activity of Fe(II)-oxidizing microorganisms results in the formation of Fe(III) (oxyhydr)oxide minerals (e.g., ferrihydrite—Fe(OH)_3_; goethite—α-FeOOH; haematite—α-Fe_2_O_3_) and, to a lesser extent, dissolved organic Fe(III) complexes (Table S1) [52,53]. Under reducing conditions, Fe(II)-Fe(III) minerals such as magnetite (Fe_3_O_4_) and green rust prevail, as well as dissolved Fe^2+^ ions or Fe(II) minerals such as siderite (FeCO_3_) or vivianite (Fe_3_(PO_4_)_2_·8H_2_O) (Table S1) [53,54]. Detailed information regarding all these different roles of microbial reactions in the iron redox cycle, which were summarized here, can be found in the reviews of Melton and his team [47] and Weber and colleagues [53]. The redox potential of different redox couples important for microbial iron cycling can vary from −0.314 V (Fe_3_O_4solid_/Fe^2+^) to +0.385 V (Fe(III)-citrate/Fe(II)-citrate) at pH 7, thus affecting the energy available for microbial carbon oxidation [55]. For example, Fe(III) oxides with relatively lower redox potential (e.g., magnetite, hematite) can provide more energy for growth, but their crystalline structure is generally less accessible for microbes [55].

Besides natural environments, iron is also abundant in several industrial wastewaters (e.g., from mining activities, metal plating, iron and steel industry) and solid wastes, due to its intensive use in the productive processes [56,57]. The iron and steel industry generates large amounts of toxic compounds, namely PAHs, cyanides, phenols, BTEX, metal fines, and dissolved metals (including iron) [58,59]. According to the World Steel Association, in 2018, the average water consumption per ton of crude steel produced was approximately 28 m^3^ [60]. Around 0.6 billion m^3^ of iron and steel industry wastewaters were discharged in Asia during 2011 [42].

Another iron-rich wastewater is acid mine drainage (AMD). AMD is acidic and mainly composed of sulfate, salts, and several metals, where iron and aluminum are present in higher amounts [61]. It is formed from the oxidation of sulfide minerals (particularly pyrite—FeS_2_) in the presence of oxygen, water, and microorganisms [62,63], by mining activities, road construction, mill tailings, and several industrial activities [63,64,65,66]. Due to its toxic characteristics, AMD is a worldwide human and environmental threat, particularly affecting underground and surface water, as well as biodiversity loss [67,68,69]. Although several studies have been performed in the last decades [68], few were conducted towards developing cost-effective procedures to manage, store, treat, and dispose of AMD.

Red mud (RM) is an important waste from mining and metallurgy activities [70,71], which is composed of several minerals (including hematite and goethite), with iron oxides accounting for approximately 30% *w*/*w* [72]. The composition of this residue can vary according to the extraction area, production, and storage processes [73]. For one ton of alumina produced, up to two tons of RM are generated. The annual global production of this waste in 2020 surpassed five billion tons [74] and around 7.6 billion m^3^ has been accumulated around the globe [72]. As a toxic industrial solid waste, due to its alkaline, chemical, and mineralogical characteristics [75], RM requires an adequate treatment before discharge. However, considering the high disposal and treatment cost (around 5% of alumina production) [76], this waste is commonly discharged in soil and groundwater systems [77]. This represents a significant impact on human health and on ecosystems, affecting productive land and groundwater, and promoting the accumulation of particles in living organisms [78]. The cytotoxic effects of iron compounds should also be considered, as at certain concentrations, they can affect living organisms. The effects of different iron forms on bacteria, algae, fish, and plants were comprehensively reviewed by Lei and colleagues [79]. However, RM can be seen as a valuable resource, instead of being considered a waste with no added value. In the last years, several studies have been performed on the recovery and potential application of RM, namely for sewage and wastewater treatment [80,81,82], polluted ecosystem remediation [83], composting [84], metal recovery [85], and particularly in the construction industry, as reviewed by Lima and colleagues [73].

Additionally, iron compounds such as ferric sulfate, ferrous sulfate, or ferric chloride (Table S1) are frequently used for organic matter and phosphorous removal in municipal and industrial wastewater treatment plants [86,87]. Therefore, iron ends up in the resultant sludge, and may also appear in low concentrations in the water line.

## 4. Anaerobic Hydrocarbon Degradation and the Effect of Iron

The iron cycling promoted by anaerobic microorganisms can directly influence the concentration of different environmental contaminants, namely those derived from industrial activities, such as hydrocarbons [53]. Therefore, the decontamination of oily waste/wastewater in anaerobic bioreactors may be improved by naturally present iron compounds or anthropogenic supplements of iron (namely by mixing different waste/wastewaters).

The microbiology of petroleum hydrocarbon degradation under anaerobic conditions was previously reviewed [88,89,90] and is concisely presented in this section. Current knowledge on these processes in the presence of iron was only briefly addressed before and is reviewed in detail here.

**Figure 3:**
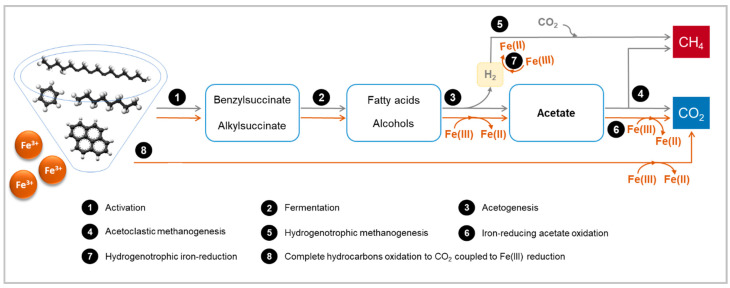
Schematic diagram of the various stages (numbers 1 to 8) potentially involved in microbial hydrocarbon conversion under methanogenic conditions (grey arrows) and in the presence of iron (III) (orange arrows).

Due to the chemical stability of petroleum hydrocarbons, the initial activation reaction (step 1—Figure 3) is often the crucial step in the degradation of these compounds [91]. Different activation mechanisms have been proposed, among which fumarate addition is the best characterized and the most widely reported, for both saturated and aromatic hydrocarbons, either under sulfate, nitrate-, metal-reducing or methanogenic conditions [91,92,93]. Fumarate addition is catalyzed by a glycyl radical enzyme named benzylsuccinate synthase (BSS) or (1-methyl)alkylsuccinate synthase (ASS/MAS). Alternative activation mechanisms include oxygen-independent hydroxylation, carboxylation, or methylation [92]. The main metabolic pathways, enzymes, and functional genes involved in anaerobic hydrocarbon biodegradation have been presented in several reviews [91,92,93,94,95,96,97] and are not addressed in this review.

The products of hydrocarbon activation (e.g., benzylsuccinate, phenol, (1-methylalkyl)succinate) are then converted by fermentative bacteria into smaller molecules, such as fatty acids and alcohols [90] (step 2—Figure 3). For example, benzoyl-CoA has been recognized as a central intermediate in the anaerobic degradation of many aromatic compounds [88], and 4- and 2-methylalkanoates have been identified during alkane degradation [98,99]. Beta-oxidation (or analogous reactions) is accepted as the metabolic pathway involved in the conversion of these intermediates [98], leading to the formation of acetate (step 3—Figure 3).

In the absence of external electron acceptors other than bicarbonate, the reactions involved in step 3 (Figure 3, grey line) are coupled to the reduction of protons, with the formation of hydrogen or formate. These reactions are only thermodynamically feasible if the end products are kept at low concentrations [90]—e.g., alkane degradation to methane is only possible at hydrogen partial pressures lower that 4 Pa [100]. This is generally accomplished by methanogenic archaea (steps 4 and 5—Figure 3), leading to the formation of biogas (mainly composed by methane (CH4) and CO2). Therefore, methanogenic hydrocarbon biodegradation is a syntrophic process that requires a close relationship between syntrophic bacteria (e.g., *Smithella*, *Pelotomaculum*) and methanogens [88,101,102].

In the presence of Fe(III) compounds, iron-reducing bacteria (IRB) may be involved in hydrocarbon degradation (Figure 1A), obtaining energy for growth via dissimilatory Fe(III) reduction. Some IRB can oxidize hydrocarbons completely to CO_2_ (step 8—Figure 3), while others convert the hydrocarbons to acetate (steps 1, 2 and 3—orange line—Figure 3), in both cases coupled to Fe(III) reduction to Fe(II) [88,103]. Most IRB are also able to grow with acetate (step 6—Figure 3), and some can use hydrogen or formate as electron donors (step 7—Figure 3) [47,103]. Thus, IRB may work as syntrophic partners of other hydrocarbon-degrading bacteria, contributing to complete hydrocarbon biodegradation [101,104]. Additionally, some IRB are capable of growing with benzoate or fatty acids with different chain lengths (long, medium, or short) [103,105,106] and hence may also play a role in the degradation of these intermediates (step 3—Figure 3). These topics are further developed in Section 5.

## 5. Fe(III) as Electron Acceptor in Anaerobic Hydrocarbon Degradation

### 5.1. Axenic Cultures Performing Hydrocarbon Degradation Coupled to Fe(III) Reduction

Hydrocarbon degradation by IRB has been reported, either in pure cultures, enrichments, or in the environment. However, IRB able to utilize *n*-alkanes are yet unknown, and only a limited number of microorganisms able to anaerobically consume aromatic hydrocarbons coupled to Fe(III) reduction has been isolated and characterized thus far (Table 2).

Among the BTEX, benzene is considered the most recalcitrant, and microbial isolates capable of anaerobic benzene degradation have been described only recently [85,104]. *Ferroglobus placidus*, a hyperthermophilic archaeon, was the first microorganism known to couple benzene oxidation to dissimilatory Fe(III) reduction, using amorphous Fe(III) oxyhydroxide as an electron acceptor (Table 2) [107]. Gene expression analysis revealed that *F. placidus* performs carboxylation of benzene to benzoate, which is the main metabolite of benzene oxidation by this microorganism [108].

Two *Geobacter* species, *Geobacter* sp. strain Ben and *G. metallireducens* GS-15T, are able to degrade benzene anaerobically coupled to the reduction of Fe(III) [109]. The stoichiometry of the reactions indicates that both species oxidize benzene to carbon dioxide [109], and for *G. metallireducens* GS-15T this was shown to occur through a phenol intermediate [110].

Regarding toluene oxidation with Fe(III) as an electron acceptor, four *Geobacter* species with this ability were isolated from environmental samples (Table 2): *Geobacter* sp. strain Ben, recovered from sediments of an oil-contaminated aquifer; *G. metallireducens* GS-15 [111,112] and two strains of *G. grbiciae*, isolated from freshwater sediments [113]; and *G. toluenoxydans* TMJ1, isolated from sludge of a monitoring well at a tar oil-contaminated aquifer [114,115]. All *Geobacter* species that are capable of degrading aromatic compounds also degrade benzoate.

**Table 2:** Overview of axenic microbial cultures able to degrade aromatic hydrocarbons coupled to Fe(III) reduction.

Substrate	Iron Compounds	Microorganism	Source/Inoculum	Notes	Ref.
Benzene	Amorphous Fe(III) oxyhydroxide	*Ferroglobus placidus*	Hydrothermal vent sediment	Optimum growth at 85 °C. Complete benzene oxidation to CO_2_. Benzoate, 4-hydroxybenzoate, and phenol also support growth. First report of an axenic Fe(III)-reducing culture degrading benzene.	[108]
Benzene Toluene	Amorphous Fe(III) oxide	*Geobacter* strain Ben	Sediments from the Fe(III) reduction zone of a petroleum-contaminated aquifer	Benzene and toluene are oxidized to CO_2_. Also degrades benzoate.	[109]
Benzene Toluene	Fe(III) citrate Amorphous Fe(III) oxide	*Geobacter metallireducens* GS-15^T^	Freshwater aquatic sediment	Benzene and toluene are oxidized to CO_2_. Also degrades benzoate, phenol, and *p*-cresol. Grows with acetate, but not with H_2_, nor formate.	[109,111,112]
Toluene	Fe(III)-citrate Fe(III)-pyrophosphate Fe(III)-NTA Amorphous Fe(III) oxide	*Geobacter grbiciae* strains TACP-2^T^ and TACP-5 (*)	Freshwater aquatic sediment	Oxidizes acetate and other Simple fatty acids, ethanol, H_2_, and formate. Also oxidizes benzoate.	[113]
Toluene	Ferrihydrite Amorphous Fe(III) oxyhydroxide Fe(III) citrate	*Geobacter toluenoxydans* TMJ1	Tar oil-contaminated aquifer	Electron recovery of 99 ± 14%. Also oxidizes acetate, benzoate, phenol, *m*- and *p*-cresol.	[114,115]
Toluene	Fe(III)-NTA	*Georgfuchsia toluolica*	Aquifer polluted with BTEX-containing landfill leachate	Toluene degradation rate of 38–40 mmol L^−1^ d^−1^.	[116]
Ethylbenzene					
Toluene	Ferrihydrite Amorphous Fe(III) oxyhydroxide Fe(III) citrate	*Desulfitobacterium* *aromaticivorans* UKTL^T^	Soil of a former coal gasification site	Complete toluene oxidation to CO_2_. Electron recovery of 93 ± 1%. It also uses acetate, benzoate, phenol, and *p*-cresol, but not H_2_.	[115]
*o*-Xylene					
Pyrene Benzo[*a*]pyrene	Fe(III) citrate	*Hydrogenophaga* sp. PYR1	PAH-contaminated river sediments	Significant pyrene and benzo[*a*]pyrene degradation.	[117]
Phenantrene	Fe(III) citrate	Anaerobic bacteria closely related to *Trichococcus* *alkaliphilus* (strain PheF2)	Mixture of petroleum-polluted soil and anaerobic sludge	100% anaerobic biodegradation of phenanthrene within 10 days of incubation	[118]

(*) Strain TACP-5 does not grow with Fe(III)-citrate.

Besides the *Geobacter* species, two other IRB were isolated that are able to degrade monoaromatic hydrocarbons under iron-reducing conditions—*Georgfuchsia toluolica*, which grows on toluene and ethylbenzene [116], and *Desulfitobacterium aromaticivorans*, which grows on toluene and *o*-xylene [115]. *Georgfuchsia toluolica* G5G6 was shown to degrade toluene and ethylbenzene at average degradation rates of about 40 μmol L^−1^ d^−1^ and 5–7 μmol L^−1^ d^−1^, respectively [119], and compound-specific stable isotope analysis (CSIA) showed that the metabolic pathways of toluene activation by this bacterium differed depending on the terminal electron acceptor [119].

PAH biodegradation under Fe(III)-reducing conditions was only reported for two axenic cultures of facultative anaerobic bacteria. *Hydrogenophaga* sp. PYR1 was isolated from PAH-contaminated river sediments and is capable of growing on pyrene and benzo[*a*]pyrene, using Fe(III) or oxygen as an electron acceptor [117]. The use of ferric citrate as the sole electron acceptor stimulated the production of a lipopeptide biosurfactant by this bacterium, which facilitated PAH degradation [117,118]. Zhang and colleagues [118] isolated a facultative anaerobic bacterium closely related to *Trichococcus alkaliphilus* (99.79% 16S rRNA gene sequence similarity), designated strain PheF2, which is able to degrade phenanthrene coupled to Fe(III) or O_2_ reduction. This was the first report of a pure culture capable of anaerobic phenanthrene biodegradation with Fe(III). This bacterium also degrades benzene, naphthalene, anthracene, pyrene, and benzo[*a*]pyrene.

Most of the known IRB involved in the oxidation of aromatic hydrocarbons are capable of utilizing various forms of soluble Fe(III) (namely ferric citrate, ferric-nitrilotriacetate (Fe(III)-NTA), or ferric-pyrophosphate) as an electron acceptor, as well as poorly crystalline Fe(III) oxide (Table 2). Regarding *Geobacter* species, effective extracellular electron transfer to insoluble Fe(III) minerals may be accomplished through various mechanisms, including microbial nanowires and c-type cytochromes [103]. The ability of *Geobacter* species to use humic substances as redox mediators in the reduction of insoluble Fe(III) oxides has also been reported [120].

### 5.2. Complex Microbial Communities Mediating Hydrocarbon Degradation Coupled to Fe(III) Reduction: Enrichment Cultures and Microcosm Studies

Current knowledge on the microorganisms involved in hydrocarbon degradation coupled to Fe(III) reduction is still limited. Laboratory-based studies have searched for information regarding the occurrence of this process in nature, as well as on the structure and dynamics of the microbial communities able to reduce Fe(III) and degrade hydrocarbons. Microcosm experiments and the establishment of enrichment cultures have been carried out, using different inocula, substrates, and iron compounds.

Only two studies report *n*-alkane degradation coupled to Fe(III) reduction. So and Young developed Fe(III)-reducing enrichment cultures able to grow with decane, dodecane, and hexadecane, although the alkane degradation proceeded at a slow rate (e.g., only 0.38 µmol of hexadecane were converted to CO_2_ per week) [121]. The inoculum used was a sediment from a highly contaminated estuary, showing that environments chronically contaminated by hydrocarbons can be a source of Fe(III)-reducing alkane-degrading microorganisms. Rizoulis and colleagues [122] used sediments from an arsenic-rich aquifer containing Fe(III) to develop microcosms supplemented with ^13^C-hexadecane. After 8 weeks of incubation, 11.2% of the sedimentary Fe(III) was microbially reduced, leading to Fe(II) formation, and 65% of the added ^13^C-hexadecane was degraded. ^13^C was incorporated into the heavy DNA fractions retrieved from the enrichments, showing that this degradation was microbially mediated, but the authors reported unsuccessful sequencing of the labelled fractions, possibly due to the low DNA concentration in the heavy DNA fraction. Bacterial community analysis was performed by 16S rRNA gene pyrosequencing and revealed an enrichment of IRB closely related to *Geobacter psychrophilus* strain P35, *Geobacter luticola*, and *Geothrix fermentans* strain H5.

Concerning BTEX and PAH biodegradation with Fe(III), an overview of most of the works reported in the literature are presented in Table 3 and Table 4. Anderson and his team [123] suggested the possible occurrence of in situ degradation of benzene, toluene, and naphthalene, based on the development of enrichment cultures from sediments of a petroleum-contaminated aquifer rich in Fe(III). These cultures were able to perform a relatively fast and almost complete mineralization of the hydrocarbons, with simultaneous Fe(II) production. Anderson and Lovley [124] showed that, contrary to what was previously thought, anaerobic benzene degradation may be widespread in nature. Kazumi and co-authors [125] reported benzene degradation coupled to Fe(III) reduction in microcosms inoculated with sediments obtained from various locations, presenting different redox conditions, contamination levels, and salinity, highlighting the benzene degradation potential of the different sediment types.

Further studies performed in microcosms inoculated with sediments from a petroleum-contaminated aquifer showed that the addition of different Fe(III) chelators, such as NTA, EDTA, phosphates, and humic acids, can increase the biodegradation rates of hydrocarbons, namely benzene and alkylbenzene (e.g., toluene), coupled to Fe(III) reduction [123,124]. This was attributed to the fact that Fe(III) is present in a wide range of hydrocarbon-contaminated sites, mainly as insoluble Fe(III) oxides, and thus chelated iron would become more bioavailable [103,126] Additionally, Jahn and his team [127] reported faster Fe(III) reduction and BTEX utilization in enrichment cultures amended with AQDS (9,10-anthraquinone-2,6-disulfonic acid). These authors developed enrichment cultures able to degrade all BTEX substrates to CO_2_, with amorphous Fe(III) oxide as an electron acceptor, and this was the first report on anaerobic degradation of *o*-xylene and ethylbenzene coupled to Fe(III) reduction. Enrichments with AQDS and benzene showed a lag phase of only 16 days, and benzene was completely degraded within 77 days, while in the absence of AQDS, a lag phase of 61 days was recorded, and complete exhaustion of benzene was attained after 162 days of incubation. Toluene and *o*-xylene degradation started immediately and was completed after 39 days in the presence of AQDS, while complete ethylbenzene degradation required 69 days of incubation with AQDS. In the absence of AQDS, degradation of ethylbenzene and *o*-xylene was much slower and did not lead to a complete depletion, even after 162 days of incubation.

Analysis of the microbial communities revealed the predominance of members of the Geobacteraceae family in benzene-degrading enriched cultures, indicating an important role of these microorganisms in the degradation of benzene coupled to Fe(III) reduction [123,127,128]. Kunapuli and colleagues [129] studied a highly enriched benzene-degrading iron-reducing culture, and proposed that an uncommon syntrophic relationship was involved in benzene degradation by this culture. DNA-SIP analysis revealed the dominance of a phylotype affiliated to the Peptococcaceae family (distantly related to cultured representatives of the genus *Thermincola*) that assimilated most carbon from the ^13^C-labeled benzene. Therefore, the Peptococcaceae phylotype seems to be responsible for the initial benzene activation and primary oxidation, since it assimilates the label more efficiently than the other two predominant phylotypes, i.e., bacteria affiliated to the Desulfobulbaceae familiy and members of *Actinobacteria*. The authors hypothesized that the Peptococcaceae phylotype transferred the electrons from benzene oxidation mainly to Fe(III), and partially to the *Desulfobulbaceae* sp., in a syntrophic relationship. The *Actinobacteria* were probably fermenting secondary carbohydrates or dead biomass.

**Table 3:** Overview of monoaromatic hydrocarbons degradation coupled to Fe(III) reduction in microcosms and enrichment cultures.

Substrate	Iron Compunds	Source/Inoculum	Community Composition	Notes	Ref.
Benzene (10 µmol kg^−1^ sediment) Toluene (10 µmol kg^−1^ sediment)	Fe(III)-NTA (2 mmol kg^−1^)	Sediments and groundwater from a petroleum-contaminated aquifer	Not analyzed	NTA adition stimulated biodegradation. No lag phases were observed after adaptation.	[125]
Benzene (10 µmol L^−1^)	Fe(III)-NTA (2 mmol L^−1^)	Sediment and groundwater from a petroleum polluted aquifer	Not analyzed	Fe-NTA stimulated biodegradation.	[130]
Benzene (3 μmol L^−1^)	Amorphous Fe(III) (10 mmol L^−1^)	River sediment	Not analyzed	After 60 days of incubation, the culture was re-fed 4 times, over which degradation was sustained and became faster.	[131]
Benzene (140 mmol L^−1^)	Fe(III) oxide (30 mmol L^−1^)	Sediment from a remote forested area contaminated by a leak in a pipeline	Enriched in members of Geobacteraceae family	Uncultivated *Geobacter* spp. seem to be related with benzene removal in this aquifer.	[127]
Benzene (900 µmol L^−1^)	Amorphous Fe(III) oxide (50 mmol L^−1^)	Soil of a former coal gasification site	3 major clone clusters: within the Clostridia (Peptococcaceae) (37%), Deltaproteobacteria (Desulfobulbaceae) (20%), and *Actinobacteria* (29%)	DNA-SIP was used to identify the microorganisms involved in benzene degradation in an iron-reducing enrichment culture.	[128]
Toluene (1 mmol L^−1^)	Fe(III) oxyhydroxide (40 mmol L^−1^)	Sediment from a tar oil-contaminated aquifer	The dominating labelled phylotype was related to the genus *Thermincola*	To ensure constantly low in situ-like concentrations, toluene was loaded in amberlite XAD7 absorber resin.	[132]
Toluene (0.96 mmol L^−1^)	Fe(III)-NTA (60 mmol L^−1^)	Contaminated tidal flat sediment	Dominant member affiliated with the *Desulfuromonas* genus	100 % toluene degradation in 35 d. DNA-SIP and metagenomic sequencing were used.	[56]
BTEX (5 mg L^−1^ each)	Amorphous Fe(III) Goethite (20 mmol L^−1^ each)	Contaminated river sediment and water	Not analyzed	All BTEX were degraded, in the following order: benzene ≤ *p*-xylene ≤ (toluene = *o*-xylene = *m*-xylene) ≤ ethylbenzene	[133]
Benzene Toluene Ethylbenzene *o*-xylene (1 mmol L^−1^ each)	Amorphous Fe(III) hydroxide (50 mmol L^−1^)	Groundwater from a tar oil-contaminated former gasworks site	Not analyzed	AQDS accelerated Fe(III) reduction and BTEX oxidation.	[134]
Benzene (20 µmol L^−1^) Toluene (100 µmol L^−1^) *o*-, *m*-, *p*-Xylene (60 µmol L^−1^ each)	Amorphous Fe(III) oxyhydroxide (10 mmol L^−1^)	Sediment and groundwater from a polluted iron-reducing aquifer	Not analyzed	Substrate swap suggested that the same group of bacteria could be involved in the removal of more than one BTEX compound. When in a mixture, benzene and toluene were degradaded simultaneouly.	[135]
Benzene (10–30 µmol L^−1^) Toluene (300–400 µmol L^−1^) *o*-, *m*-, *p*-Xylene (300–400 µmol L^−1^ each)	Amorphous Fe(III)	Sediment and groundwater from a polluted iron-reducing aquifer	Not analyzed	BTX degradation rates in enrichments progressively increased in time.	[136]
BTEX (15 mg L^−1^)	FeCl_3_ (3.58 mmol L^−1^)	Pristine sediment and groundwater collected from a shallow well	Not analyzed	BTEX and trimethylbenzene isomers were degradaded in microcosms containing both nitrate and Fe(III).	[137]
BTEX (100 mg L^−1^)	Goethite Akaganeite (0.1 g L^−1^ each)	Contaminated aquifer	Concentration not mentioned	BTEX removal was higher with akaganeite (46%, 58%, 59%, and 70 % for benzene, toluene, ethylbenzene, and xylenes, respectively).	[138]
BTEX (concentration not determined)	Fe(III)-NTA (5 mmol L^−1^)	Groundwater sample from a BTEX-contaminated aquifer (leakage of a petrol station)	Enriched in *Geobacter*-related bacteria and a *Rhodoferax* phylotype	In the laboratory, *Rhodoferax*-related bacteria were not enriched. *Geobacter* was readly enriched, but the diversity of BSS gene, both in the enrichments and in the initial groundwater sample, suggested that *Geobacter* was not a key player in toluene degradation in this site.	[128]

**Table 4:** Overview of PAH degradation coupled to Fe(III) reduction in microcosms and enrichment cultures.

Substrate	Iron Compunds	Source/Inoculum	Community Composition	Notes	Ref.
[C^14^]Naphthalene (1 µCi)	Fe(III) oxide (9.6 µmol g^−1^)	Sediments from petroleum-contaminated aquifers	Not analyzed	After 85 days of incubation, around 90% naphtalene degradation.	[124]
Naphthalene Acenaphthalene Phenanthrene Anthracene Pyrene Fluoranthene (above solubility concentration)	Ferrihydrite	Coal tar-contaminated sediment from a former coal gasification plant	Not analyzed	PAH solubility was enhanced by hydroxypropyl-β-cyclodextrin (HPCD) concentrations up to 5 g L^−1^. Low HPCD concentrations (0.05–0.5 g L^−1^) also enhanced phenanthrene mineralization by 25%. The culture was still able to mineralize PAHs at 10 °C.	[139]
Naphthalene (2 mmol L^−1^)	Ferrihydrite (50 mmol L^−1^)	Sediment from an aquifer contaminated with tar oil	Enriched in bacteria belonging to the Peptococcaceae family	7.5 ± 3 µmol naphthalene degraded after 180 days. Also grows with 1- and 2-methylnaphthalene.	[140]
Phenanthrene (20–30 mg L^−1^)	Ferric citrate (20 mmol L^−1^)	Petroleum-contaminated soil + coking sludge + domestic sludge (5:1:1 as volatile suspended solids)	Bacterial community: Carnobacteriaceae (18%), Geobacteraceae (9%), Anaerolinaceae (9%) Archaeal community: Methanobacteriaceae (28%), Methanosarcinaceae (14%)	At the end of the enrichment process (244 days), phenanthrene degradation rate stabilized at 2.7 μmol L^−1^ d^−1^.	[141]

Besides benzene, this syntrophic community was able to grow on phenol, benzoate, and 4-hydroxybenzoate, but not on toluene, ethylbenzene, or xylene isomers as the sole carbon source [129]. The enzymes involved in benzene oxidation by this culture were further studied through a combined proteomic and genomic approach [142]. The authors proposed that the identified gene sequences are constituents of a putative benzene degradation gene cluster, composed of carboxylase-related genes, which probably catalyze the initial activation reaction in benzene degradation by this culture.

Within the Peptococcaceae family, relatives of the genus *Thermincola* were identified by Pilloni and colleagues [132] as iron-reducing toluene degraders, in microcosms prepared with sediment from a tar oil-contaminated aquifer [127]. ^13^C-toluene and amorphous Fe(III) oxyhydroxide were used as the electron donor and acceptor, respectively. In this study, DNA stable-isotope probing (DNA-SIP) was combined with high-throughput pyrosequencing of amplicons from SIP incubations.

DNA-SIP and metagenomics sequencing were also used by Kim and colleagues to study toluene degradation by IRB [143]. ^13^C-toluene degradation and close-to-stoichiometric ^13^C-CO_2_ production were verified in the microcosms experiments, as well as concomitant Fe(II) formation. The microbial community was dominated by members of the *Desulfuromonas* genus. The reconstruction of the metabolic pathways from the *Desulfuromonas* sp. genome demonstrated its metabolic versatility for degrading aromatic hydrocarbons and utilizing different electron acceptors.

The potential of using insoluble Fe(III) oxide to stimulate hydrocarbon degradation in petroleum-contaminated harbor sediments, in which sulfate reduction was the terminal electron-accepting process, was investigated by Coates and colleagues [144]. The authors proposed that the addition of Fe(III) could switch the electron flow from sulfate to Fe(III) reduction. Benzene, toluene, and naphthalene were tested, but hydrocarbon degradation was not stimulated by Fe(III), most probably due to the reduced number of IRB originally present in the sediments studied. Similar results were also obtained by Li and his team [145] when studying the effect of Fe(III) on the anaerobic biodegradation of a mixture of PAHs (fluorene, phenanthrene, fluroanthene, and pyrene) in mangrove sediment slurries [145]. Fe(III) amendment did not significantly affect PAH biodegradation, nor microbial population sizes (as indicated by most probable number, MPN). This was attributed to the abundance of sulfate and nitrate in the sediment, which were possibly used by the indigenous microorganisms, even in the groups amended with Fe(III).

### 5.3. Complex Microbial Communities Mediating Hydrocarbon Degradation Coupled to Fe(III) Reduction: Sediment Columns and Field Studies

Several limitations have been associated with laboratory assays, e.g., their relevance in the representation of the natural conditions and processes, difficulties in collecting representative samples of the subsurface microbial communities, due to their inherent heterogeneity, as well as potential disturbance of the microbial communities by the sampling, preservation, and laboratory incubation procedures [146]. Therefore, in order to mimic environmental conditions, and to study the microbial physiology, ecology, and interactions involved in anaerobic hydrocarbon degradation in a more realistic way, laboratory soil/sediment columns or field studies (e.g., in situ microcosms, push–pull tests, and tracer tests) have been performed. The studies utilizing such methodologies applied to hydrocarbon biodegradation under iron-reducing conditions are described in this section.

Langenhoff and colleagues [147] studied the biodegradation of a mixture of naphthalene, toluene, and benzene (25 μmol L^−1^ individual concentrations) under iron-reducing conditions by using sediment columns. The columns were filled with of a mixture of anaerobic soil, PAH-contaminated sediment, and granular sludge. The hydrocarbon mixture was continuously fed, whilst amorphous Fe(III) oxide was mixed through the column material (approximately 5 mmol) and re-added upon depletion. Naphthalene and benzene were not degraded, but toluene degradation increased, leading to an undetectable concentration of this hydrocarbon in the effluent after about 2 months of operation. Another sediment column experiment was performed by Zheng and his team [148], for the analysis of intrinsic toluene biodegradation coupled to microbial Fe(III) reduction in a polluted aquifer. Columns were packed with contaminated aquifer sediment, and ferric iron was used as an electron acceptor. Biodegradation of toluene coupled to Fe(II) production was observed, and slower pore water velocity led to a higher biodegradation rate.

Enhanced degradation of phenanthrene and pyrene was reported by Yan and colleagues [149] in sediment microbial fuel cells amended with amorphous ferric hydroxide (0.01 g g^−1^, relative to sediment wet weight). Sediment column bioreactors were set up, electrodes composed of stainless steel cylinders were installed, and a fixed external resistance of 100 Ω was applied. PAHs were removed mainly within the first 22 days of experiments, at degradation rates of 0.0836 d^−1^ and 0.1363 d^−1^ for phenanthrene and pyrene, respectively. After this initial period, their concentration decreased slowly but steadily, reaching 99.5 ± 0.2% and 94.8 ± 0.6% removal for phenanthrene and pyrene, respectively, after 240 days. In this system, Fe(III)-reducing activity was three times higher than in the natural attenuation control, and the phylogenetic analysis indicated that many clones retrieved from the anode biofilm corresponded to dissimilatory metal-reducing bacteria, namely *Geobacter*. The authors conclude that PAH removal in this system was due to the combination of electrode-reducing processes and other anaerobic redox pathways that coexisted in the sediment and were not mutually exclusive. Similar results were obtained by Yu and his team [150] in soil microbial fuel cells (SMFC) prepared with PAH-contaminated soil from a petrochemical industrial zone. The removal rates of anthracene, phenanthrene, and pyrene were higher in the closed rather than open SMFC and were accompanied by electricity generation. The microbial community at the anode surface was dominated by bacteria belonging to the *Geobacter* genus.

Regarding field studies, a cross-section of a crude oil-contaminated aquifer was monitored by Bekins and colleagues [151], to analyze the effect of hydrocarbon contamination on microbial activity and distribution. Samples were collected horizontally from the source of the contamination plume to 66 m down-gradient, and vertically from above the water table to the base of the plume. It was possible to outline a map of defined physiological zones, with iron-reducing microorganisms representing one of the most abundant microbial communities in the contaminated area. Müller and his team [152] tested a novel strategy to enhance in situ biodegradation of BTEX and PAHs. This strategy consisted in the combined stimulation of iron and sulfate reduction processes as terminal electron acceptors for the biological removal of these contaminants. For that, a sustainable and low-cost product, retrieved from acid mine drainage, was used, which contained 88.3% *w*/*w* iron oxide (goethite) and 1.7% total sulfates. Acetate was also supplemented, to promote the initial growth of the hydrocarbon-degrading Fe(III)-reducing microorganisms. Lower levels of benzene and naphthalene were always detected in the biostimulated experiment, relative to the natural attenuation site, showing that the combined addition of iron and sulfate enhanced the degradation of aromatics. An increase in the dominance of members of the *Geobacter* genus and of the Thermodesulfovibrionaceae family was detected, indicating their potential role in aromatic hydrocarbon degradation.

Cozzarelli and colleagues [146] measured the degradation rates of BTEX and eight alkyl-benzene isomers over a time period of three years, in a crude oil-contaminated aquifer. An in situ microcosm was set up within a well-defined Fe(III)-reducing zone, which allowed the encasement of a small region of the aquifer. The in situ microcosms were perfused with native groundwater and could be amended with the aromatic compounds of interest without disturbing the indigenous microbial populations. Microorganisms of the *Geobacter* cluster were previously shown to be enriched in the sediments from this location [130]. Biodegradation of the BTEX compounds followed the order: toluene ≥ *o*-xylene > *m*-, *p*-xylenes > benzene > ethylbenzene. Benzene and ethylbenzene degradation was preceded by a long lag phase (~200 days), and threshold concentrations, below which no degradation occurred, were identified for these compounds. In situ microcosm systems have also been used by several authors to study the degradation of aromatic and chlorinated aliphatic hydrocarbons coupled to Fe(III) reduction in landfills [153,154,155].

Winderl and his team [156] performed a high-resolution monitoring of the microbial community present in a tar oil-contaminated aquifer, covering between 6 and 13 m depth below ground surface. Underneath the contaminated plume core, distinct biogeochemical gradients could be identified in the centimeter scale, and a highly specialized toluene-degrading microbial community was found, mainly composed of bacteria related to the *Geobacter* and *Desulfocapsa* genera (which are known iron and sulfate reducers, respectively). The results obtained reinforce the hypothesis previously stated by Bauer and co-workers [157] that plume fringes are the actual spots of contaminant degradation, and highlight the close relation between redox processes, contaminant degradation, and microbial distribution in contaminated aquifers.

### 5.4. Fe(III) as Electron Acceptor in the Degradation of Intermediates of Anaerobic Hydrocarbon Conversion

As mentioned in Section 4 and shown in Figure 1 and Figure 3, it is possible that IRB may also have a role in the complete degradation of hydrocarbons by working as syntrophic partners (consuming acetate or hydrogen/formate), or by utilizing intermediary products of hydrocarbon conversion. The continuous consumption of the intermediates most probably facilitates the chain of reactions involved in hydrocarbon degradation. Long-chain fatty acids (LCFAs) have been appointed as potential intermediates of aliphatic hydrocarbon degradation, and some IRB are capable of growing with LCFAs, e.g., *Desulfuromonas palmitatis* [103] and *Geothrix fermentans* [103]. Oleic acid degradation was stimulated by lepidocrocite and ferric-NTA in batch assays, i.e., mineralization reached 98% and 67%, respectively, relative to 58% in unamended incubations [158]. Li and his team [159] reported faster canola oil biodegradation in the presence of ferric hydroxide. A syntrophic relationship between bacteria from the *Syntrophomonas* and *Geobacter* genera was also described during oleate (unsaturated C18 LCFA) degradation in the presence of Fe(OH)_3_ [160]. Regarding the intermediaries of aromatic hydrocarbon degradation, some IRB are reported to grow with phenol or benzoate coupled to Fe(III) reduction (see Table 2 and Section 5.1 and Section 5.2 for examples). Tang and colleagues reported faster phenol degradation and methane production in the presence of Fe(III) citrate or poorly crystalline ferrihydrite, relative to control assays without Fe(III) oxides. IRB such as *Trichococcus* and *Caloramator* species were enriched by Fe(III) citrate, and these bacteria were possibly involved in phenol or benzoate degradation into fatty acids via dissimilatory Fe(III) reduction. Further degradation of the metabolic intermediates most probably occurred through syntrophic interactions with *Methanothrix* species [161]. Similar results were obtained by Li and his team [162], who reported a 1.3-fold increase in the phenol degradation rate, as well as higher cumulative methane production, due to the simultaneous addition of citrate and sub-stoichiometric Fe(OH)_3_ amounts, relative to assays amended only with Fe(OH)_3_. Citrate increased the solubility and consequent bioavailability of Fe(OH)_3_ and lowered the reduction potential of Fe(III)/Fe(II), promoting the enrichment of IRB that most probably proceeded syntrophic metabolism with methanogens.

Using a contaminated soil from a former coal gasification site as the inoculum, Marozava and colleagues reported the enrichment of an anaerobic culture able to degrade naphthalene, 1-methylnaphthalene, and 2-methylnaphthalene using Fe(OH)_3_ as terminal the electron acceptor. By performing SIP and metagenome analysis of the culture grown with fully labeled ^13^C-naphthalene, the authors revealed the presence of mainly two bacteria related to the Thermoanaerobacteraceae and Desulfobulbaceae families. The labeled carbon was mostly incorporated by the Thermoanaerobacteraceae species, and putative genes involved in naphthalene degradation were identified in the genome of this organism via assembly-based metagenomics. Therefore, the authors suggested that these bacteria degraded PAHs and excreted electrons, e.g., as hydrogen or 3,4-dihydroxybutanoic acid (which was detected in the culture supernatant), which were further oxidized by the Desulfobulbaceae species coupled to Fe(III) reduction [163].

Ferrihydrite was also shown to accelerate hexadecane-dependent methanogenesis [164]. The methanogenesis rate was 2.3 times higher in microcosms amended with iron and hexadecane, comparative to the controls (without an added electron acceptor). An increase of *Methanosarcina mcrA* gene copies, and the fact that ferrihydrite addition did not trigger the growth of Geobacteraceae or other IRB, suggests that *Methanosarcina* species performed Fe(III) reduction (which concurs with the report of van Bodegom and colleagues [165]. Proteobacteria members able to degrade hydrocarbons were also identified, reinforcing that the direct positive effect of Fe(III) on methanogenesis indirectly enhanced bacterial degradation of hexadecane.

## 6. Iron as a Catalyst in Anaerobic Hydrocarbon Degradation

### 6.1. Effect of Iron-Based Conductive Materials in Interspecies Electron Transfer

In the last years, conductive materials (CMs) have been reported to enhance methane production by anaerobic microbial communities in various ecosystems [18]. This effect has been ascribed to the so-called conductive particle-mediated interspecies electron transfer (CIET), a process that may occur as an alternative or as a complement to direct interspecies electron transfer, or to indirect electron transfer via hydrogen or formate [166]. Interspecies electron transfer (IET) is crucial for the syntrophic conversion to methane of diverse substrates (e.g., hydrocarbons) or their key intermediates (e.g., butyrate, acetate, benzoate), as mentioned in Section 4.

Fe(III) oxides with a relatively high electrical conductance, such as magnetite, were reported to promote CIET between syntrophic partners in methanogenic communities, as well as other iron-containing materials such as RM, stainless steel, and waste iron scraps [167,168,169]. Moreover, iron-based CMs may take the place of outer membrane c-type cytochromes (OmcS), acting as electron conduits and forming an electrically conductive network which assists the long-distance extracellular electron transport [165].

Carbon-based CMs, such as graphene oxide and biochar, have been reported to promote the biodegradation and the electrochemical activity of anaerobic cultures that degrade petroleum hydrocarbons [170]. The effect of iron-based CMs on the anaerobic degradation of hydrocarbons or their intermediate compounds (Figure 1B) was only recently addressed by a few authors. For example, Ye and his team [171] used magnetite as an iron-based CM and evaluated its effect on phenanthrene degradation by a methanogenic community enriched from petroleum-contaminated soil. Phenanthrene degradation and methane production rates were improved by 26% and 22%, respectively, in the presence of the magnetite, but no significant effect was verified on the relative abundance of methanogens in the enrichments. By inhibiting the methanogens with 2-bromoethanesulfonate, the authors were able to verify that syntrophic cooperation between bacteria and methanogens was necessary for complete phenanthrene degradation and pointed to the occurrence of CIET promoted by magnetite.

Similar results were verified by Tang and colleagues [161] during methanogenic phenol degradation. These authors reported that the presence of magnetite and hematite resulted in high electron recovery efficiencies (i.e., 93–97% and 86–89%, respectively) and stimulated the growth of IRB such as *Shewanella* and *Enterococcus*. It was suggested that magnetite served as an electron conduit, facilitating IET between *Enterococcus* and *Methanothrix* species in the syntrophic degradation of phenol intermediates, such as fatty acids, to methane. Enhanced phenol degradation by magnetite was also reported by Yan and colleagues [172], mainly due to the role of magnetite as an electron conduit, but also to the enrichment of certain extracellular polymeric substances (EPSs), such as proteins and humic substances, which can act as electron shuttles, benefiting IET. In this work, the addition of magnetite enriched phenol-degrading bacteria (e.g., *Syntrophorhabdus, Syntrophus*), as well as methanogens assigned to *Methanosaeta*.

The addition of conductive iron oxides (hematite and magnetite) enhanced the anaerobic degradation of benzoate, both under methanogenic and sulfate-reducing conditions [173,174]. Under methanogenic conditions, 89–94% of the electrons released from benzoate were recovered as methane, and the methane production rates were 25% and 53% higher with hematite and magnetite, respectively, than in the controls without iron oxides [173]. Under sulfate-reducing conditions, benzoate degradation rates were enhanced 82% and 92% with hematite and magnetite, respectively, compared with the controls, and increased with magnetite concentrations. Microbial reduction of iron oxides only accounted for 2% to 11% of electrons produced by benzoate oxidation [174]. In both studies, acetate was detected as an intermediate product, implying the occurrence of syntrophic benzoate degradation. Therefore, the stimulatory effects of the iron oxides on benzoate degradation were probably associated to CIET between syntrophic bacteria and methanogenic [173]) or sulfate-reducing partners [174]. Aromokeye and colleagues [175] also reported accelerated benzoate degradation coupled to enhanced methanogenesis, which occurred concurrently with Fe(III) reduction, in incubations with marine sediments and crystalline iron oxides (i.e., magnetite and hematite). Therefore, crystalline iron oxides acted as conduits for direct electron transfer, and simultaneously as electron acceptors. In contrast, Fe(III) reduction was the main pathway in the incubations with poorly crystalline lepidocrocite, inhibiting methanogenesis, as well as benzoate degradation. Novel bacteria, belonging to the *Thermincola* and *Dethiobacter* genera (phylum Firmicutes), *Melioribacter* (phylum Ignavibacteria), and Deltaproteobacteria bacterium SG8_13 (phylum Proteobacteria, family Desulfosarcinaceae) were identified as capable of degrading benzoate in marine sediments.

Magnetite was also reported to enhance pollutant removal and methane production during Fischer–Tropsch wastewater treatment [176]. These wastewaters are characterized by high chemical oxygen demand (COD) values, generally up to 30 g L^−1^, mainly consisting of alcohols, monocarboxylic organic acids, and hydrocarbons (typically alkanes and alkenes) [177]. Anaerobic sequential batch reactors were operated to treat a raw Fischer–Tropsch wastewater (COD of ~31 g L^−1^) over a period of 240 days, in the presence of different magnetite dosages (0–0.6 g). The optimum magnetite dose (0.4 g) resulted in a COD removal efficiency of 84 ± 2% and in a cumulative methane production 49% higher than in the absence of the conductive material. Lower CO_2_ and hydrogen concentrations pointed to the occurrence of magnetite-facilitated CO_2_ reduction to methane.

A novel three-dimensional mesh magnetic loofah sponge biochar (magnetized with Fe_3_O_4_) was applied to remediate PAH-contaminated sediment [178]. Compared to other carbon-based materials, this magnetic biochar achieved the highest PAH removal, mainly due to its adsorption capacity and biostimulation. Microorganisms associated with aromatic hydrocarbon degradation were specifically enriched, and methanogenesis became the main electron-accepting process. The biostimulation effect of this material was shown to be closely related with its superior conductive property, relative to the other carbon-based materials tested. The beneficial effects of biochar are also extended to AD by improving biogas desulfurization and bioenergy recovery [179], thus contributing to the economic and environmental progress.

Lin and colleagues [180] investigated the performance of AD reactors containing a bioelectrode system, operated with a voltage of 0.8 V applied from an external power supply (microbial electrolysis cell, AD-MEC), for the treatment of phenanthrene in wastewater sludge. Metallic cathodes composed by stainless steel mesh (SSM) and titanium mesh (TM) enhanced phenanthrene degradation and methane production relative to the control, which was a typical AD reactor (without electrodes). In particular, phenanthrene removal and cumulative methane production in the SSM reactor were 40% and 19% higher, respectively, than in the control. Analysis of the microbial communities’ showed that *Geobacter* spp. were enriched on the anode biofilms and were absent in the control reactor. Members of the *Geobacter* genus are known to be electrochemically active and able to transfer electrons to electrodes, as well as capable of PAH degradation [150]. Additionally, the high relative abundance of *Methanobacterium* in the SSM and TM reactors suggested that AD-MEC with metallic cathodes promoted the release of hydrogen, enhancing CO_2_ reduction to methane. The SSM reactor produced around 10% more methane than the TM reactor, which was ascribed to the etching of the SSM electrode during digestion, which may have separated iron into the system. Iron may have been used as the electron acceptor, thus contributing to the enrichment of *Geobacter* spp.; zero-valent iron (ZVI) has been previously shown to increase the methane yield in AD systems [16,50].

### 6.2. Effect of Zero-Valent Iron (ZVI) in Anaerobic Hydrocarbons Degradation

Zero-valent iron (ZVI) has been widely studied and applied to enhance the degradation of refractory organic compounds in wastewater, groundwater, and contaminated soils. ZVI undergoes several possible reactions (see the reviews [16,17] for details) from which hydroxide radicals can be formed and work as an oxidant to increase contaminants’ biodegradability and removal. Alternatively, ZVI can donate electrons and reduce organic compounds, such as halogenated hydrocarbons (e.g., trichloroethylene, dichloroethylene, vinyl chloride, 1,2-dichloroethane), resulting in the oxidation of Fe^0^ to Fe(II) [16,181]. Most studies on hydrocarbon removal with ZVI in AD processes are focused on halogenated hydrocarbons (reviewed by Li and colleagues [15], and the toxicity of these compounds was generally reduced [15,182].

In AD systems, the enhancement of methane production by nano- and microscale ZVI (nZVI and mZVI) has also been frequently reported and was recently reviewed in different works [16,17,50]. However, the occurrence of ZVI-enhanced methanogenic hydrocarbon degradation (Figure 1D) is only poorly explored. The positive effect of ZVI on methanogenesis has been ascribed to several mechanisms (see the reviews of Tian and Yu [158] and Li and colleagues [17]). Briefly, the anaerobic iron corrosion donates electrons that can be used directly for biological CO_2_ reduction or can lead to the formation of H_2_, enhancing the abundance and activity of H_2_-consuming microorganisms, namely hydrogenotrophic methanogens. For example, Zhu and his team [183] showed that the addition of ZVI shavings enhanced hydrogen production. Accelerated CO_2_ reduction to methane by ZVI was reported by Ma and colleagues [184] in enrichment cultures developed from oil reservoir PW. The archaeal community in the ZVI-amended enrichment cultures was dominated by thermophilic hydrogenotrophic methanogens belonging to the *Methanothermobacter* genus. By coupling magnetite and ZVI, the AD of phenol was increased [185]. ZVI improved the growth of hydrogenotrophic methanogens and magnetite enhanced the growth of syntrophic acetate-oxidizing bacteria (*Clostridium* spp.), which interacted in syntrophy, thus contributing to the observed synergistic effect on phenol degradation [186].

Due to its reductive character, ZVI also decreases the oxidation reduction potential (ORP), inducing a more favorable environment for the activity of anaerobic microorganisms. For example, ZVI addition significantly enhanced the removal of COD, phenolics, and nitrogen-containing heterocyclic compounds in the anaerobic treatment of coking wastewater [187]. By decreasing ORP, ZVI distinctly altered the microflora structure and enriched functional microbes involved in the anaerobic degradation of the aromatic pollutants.

Synergistic interactions between nZVI and different functional microbial groups have also been studied and were presented in different reviews [49,181,186,188]. In this approach, nZVI acts as a reducing agent in the conversion of higher halogenated compounds into lower halogenated organics, which are further degraded by organohalide-respiring bacteria (e.g., *Dehalococcoides*). Additionally, nZVI corrosion influences the ORP and generates hydrogen, as was previously mentioned, thus creating conditions that stimulate the abundance and activity of organohalide-respiring bacteria, which are able to synergistically degrade organohalides. As such, coupling abiotic and biotic degradation processes led to a more efficient degradation of halogenated hydrocarbons. Similar results were obtained by Wu and colleagues [186] with mZVI in a field study. These authors demonstrated that mZVI coupled with biostimulation was an effective method to promote the degradation of chlorinated aliphatic hydrocarbons in contaminated groundwater. Nevertheless, it is important to highlight that toxic effects may occur, depending on the ZVI species and dosage. Coupling of nZVI with IRB is another possible approach to enhance hydrocarbon degradation, since IRB can reduce the iron (hydr)oxides, which were formed on the surface of nZVI during its chemical corrosion, to ferrous iron compounds, therefore solubilizing the precipitate layers and reactivating the passivated nZVI [49,189].

## 7. Indirect Roles of Iron in Anaerobic Hydrocarbons Degradation

Magnetic iron-based nanomaterials can be used as hydrocarbon adsorbents (Figure 1C), with the potential to reduce the toxicity of these compounds towards anaerobic microorganisms and indirectly enhancing its biodegradation [190]. Magnetic nanoparticles (magnetite Fe_3_O_4_) can be simply recovered after use by the application of a magnetic field. These nanoparticles present a high surface area, but they easily aggregate, which decreases the removal efficiency. To overcome this limitation, surface modifications have been applied, namely by coating the surface of Fe_3_O_4_ particles with multiwalled carbon nanotubes [191], carbon [192], SiO_2_-graphene [193], and polyaniline [194], with successful applications in the removal of PAHs. For example, Fe_3_O_4_@polyaniline presented a good adsorption capacity for fluoranthene, pyrene, and benzo[*a*]pyrene from environmental water samples and could be reutilized up to 20 times with consistently good adsorption efficiencies [194]. Sarcletti and colleagues [195] functionalized commercial Fe_3_O_4_ nanoparticles with hexadecylphosphonic acid, to render them superhydrophobic and superoleophilic. These nanoparticles were able to extract single hydrocarbons (such as alkanes and aromatics) from water, as well as complex hydrocarbon mixtures, up to 14 times the sorbent volume. The sorbent material maintained the extraction rate over 10 consecutive extraction cycles. Magnetic shell cross-linked knedel-like nanoparticles (MSCK) were constructed, using magnetic iron nanoparticles, for hydrocarbon sequestration from crude oil, and were capable of removing hydrocarbons up to 10 times their own weight [196]. Once loaded, these nanoparticles were easily recovered by applying an external magnetic field.

Another indirect effect of iron was reported by Beller and colleagues [197] in the degradation of toluene coupled to sulfate reduction. The addition of millimolar concentrations of amorphous Fe(OH)_3_ facilitated the onset of toluene degradation and accelerated the degradation rate. This positive effect was attributed to secondary abiotic reactions between Fe(III) and H_2_S, thus decreasing the toxicity of the latter towards the toluene-degrading bacteria.

## 8. Knowledge Gaps and Future Perspectives

Different iron species and iron-containing nanomaterials have a high potential to mitigate hydrocarbon pollution in natural and engineered environments, mainly by acting as electron acceptors, as electron transfer mediators, or as electron donors for enhanced methane production. Significant progress has been made in the past decades regarding the identification of the microorganisms involved in the degradation of petroleum-based alkanes, monoaromatics, and PAHs, directly or indirectly coupled to the reduction of soluble and less soluble Fe(III) compounds. Nevertheless, despite these advances, current knowledge on the microbial diversity, interactions, and mechanisms responsible for these transformations is still scarce and is still far from being accurately understood. This knowledge gap is even more evident when considering the novel strategies of conductive iron-containing nanomaterials and ZVI addition, or the use of novel reactor configurations such as microbial electrolysis cells.

Multi-omics technologies can contribute to a better understanding of the metabolic pathways through the identification of relevant genes and enzymes, further advancing the comprehensive knowledge on this topic. As such, metagenomic and metatranscriptomic studies are important to unveil the roles of different iron species/materials on the microbial ecology in anaerobic hydrocarbon degradation processes. These may pave the way for the development of novel treatment strategies, targeting a more efficient management of the microbial activities towards increasing hydrocarbon biodegradation. Currently, the addition of conductive iron oxides or ZVI are forefront strategies for enhancing the anaerobic conversion of hydrocarbons to methane. Furthermore, the combination of abiotic processes (e.g., adsorption; ZVI as reducing agent) with biological degradation also presents a high potential for research and development.

The addition of different iron compounds to an extended range of complex substrates, including hydrocarbon-contaminated wastes, must be considered, since the AD process performance can potentially be improved, resulting in higher energy recovery rates through biomethane production. This approach could also translate into the better economic performance of anaerobic digesters.

In general, the complexity of the microbial systems, as well as of the hydrocarbon mixtures in crude oil, the possible occurrence of iron speciation, and the fact that the microbial processes are generally slow, constitute important obstacles to the research, development, and innovation in the field. Moreover, most studies were performed at laboratory scale and, therefore, pilot-scale and full-scale experiments are lacking. The effect of the different iron forms should also be studied in continuous systems, to assess its medium/long-term impacts, as well as the optimum iron dosage that maximizes the system’s performance, while avoiding its overload. Considering nanomaterials, stringent monitoring and control strategies should be implemented.

In conclusion, the use of iron compounds to assist anaerobic hydrocarbon degradation still requires further research efforts to achieve efficient and sustainable processes that are technically and economically feasible and can be further translated into the market.

## Figures and Tables

**Table 1:** Typical ranges of saturated and aromatic hydrocarbons in produced water (PW).

Saturated Hydrocarbons	BTEX	PAH	Ref.
-	0.068–578 mg L^−1^	40–3000 μg L^−1^	[33]
-	0–48 mg L^−1^	-	[34]
17–30 mg L^−1^	0.39–35 mg L^−1^	-	[35]

## Data Availability

Not applicable.

## Supplementary Materials

The following supporting information can be downloaded at: https://www.mdpi.com/article/10.3390/microorganisms10112142/s1, Table S1: Chemical formula, structure and aspect of various iron compounds.
