# Supplementary material for: Iron Compounds in Anaerobic Degradation of Petroleum Hydrocarbons: A Review
Source: Microorganisms. 2022 Oct 29;10(11):2142. doi: 10.3390/microorganisms10112142 (PMC9695802; doi:10.3390/microorganisms10112142)
Supplement: Supplementary file 1 [file microorganisms-10-02142-s001.zip › microorganisms-1975533-supplementary.pdf]

Iron compounds in anaerobic degradation of petroleum hydrocarbons: a review

Ana R. Castro [1,2], Gilberto Martins [1,2], Andreia F. Salvador [1,2], and Ana J. Cavaleiro [1,2*]

[1] CEB—Centre of Biological Engineering, University of Minho, 4710-057 Braga, Portugal
[2] LABBELS—Associate Laboratory, 4704-553 Braga/Guimarães, Portugal
* Correspondence: acavaleiro@deb.uminho.pt; Tel.: +351253604423

Table S1. Chemical formula, structure and aspect of various iron compounds

Iron compounds	Chemical formula	Crystal structure	Appearance
Akaganeite	β-FeOOH	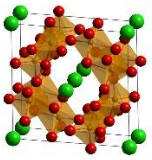 https://som.web.cmu.edu/structures2/S092-Akaganeite.html	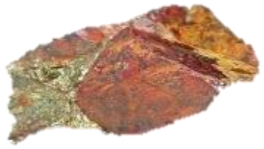 https://assignmentpoint.com/akaganeite-properties-and-occurrence/
Feroxyhyte	δ-FeOOH	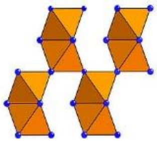 https://commons.wikimedia.org/wiki/File:Structure_ferrihydrite.jpg	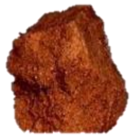 http://www.msrblog.com/science/geographic-minerals/feroxyhyte.html
Ferrihydrite	$Fe(OH)_3$	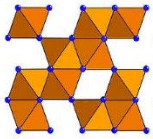 https://commons.wikimedia.org/wiki/File:Structure_ferrihydrite.jpg	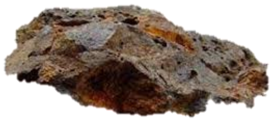 http://msrblog.com/assign/science/geographic-minerals/ferrihydrite.html
Goethite	α-FeOOH	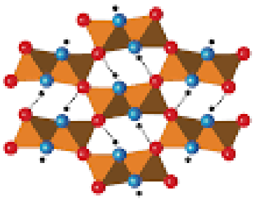 http://www.diva-portal.org/smash/get/diva2:621003/FULLTEXT01.pdf	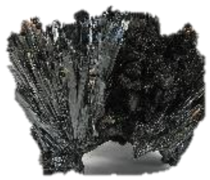 https://en.wikipedia.org/wiki/Goethite
Hematite	α-Fe_2O_3	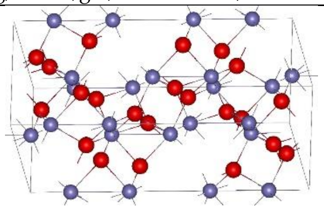 https://en.wikipedia.org/wiki/Hematite#/media/File:Hematite_structure.jpg	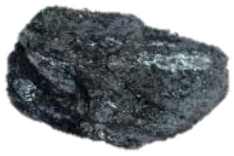 https://www.fishersci.com/shop/produ

Iron compounds	Chemical formula	Crystal structure	Appearance
			cts/hematite-mineral-specimen-6/p-7203007
Iron(II) chloride	$FeCl_2$	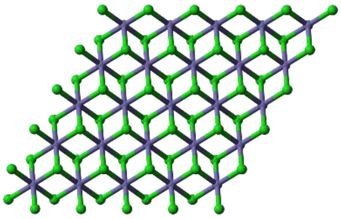<br>https://en.wikipedia.org/wiki/Iron%28II%29_chloride	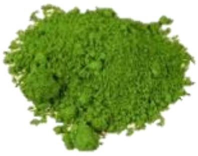<br>https://www.alibaba.com/product-detail/Ferrous-chloride-tetrahydrate-with-FeCl2-4_1600261422277.html
Iron(III) chloride	$FeCl_3$	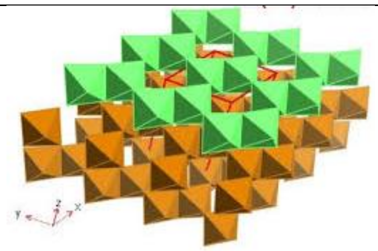<br>https://www.webelements.com/compounds/iron/iron_trichloride.html	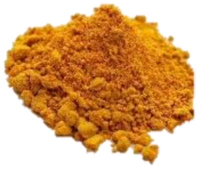<br>https://www.exirco.com/news/22/Ferric%20chloride%28solid%29?lang=en
Iron(II) sulfate	$FeSO_4$	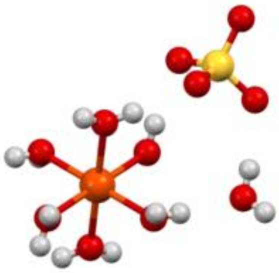<br>https://en.wikipedia.org/wiki/Iron%28II%29_sulfate	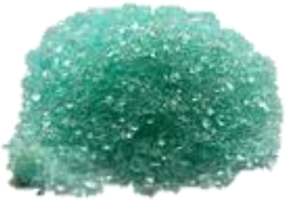<br>https://en.wikipedia.org/wiki/Iron%28II%29_sulfate
Iron(III) sulfate	$Fe_2(SO_4)_3$	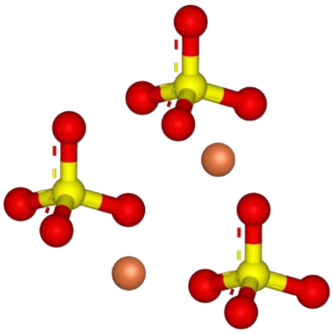<br>https://commons.wikimedia.org/wiki/File:Iron%28III%29-sulfate-3D-balls-ionic.png	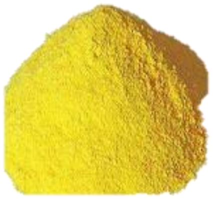<br>https://mubychem.net/ferric-sulfate.php
Maghemite	γ-Fe_2O_3	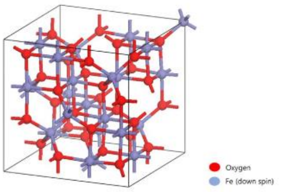<br>https://www.researchgate.net/publication/299510704_Theoretical_studies_on_structural_properties_of_Iron_Oxides/figures	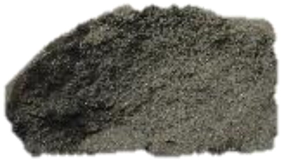<br>https://en.wikipedia.org/wiki/Maghemite

Iron compounds	Chemical formula	Crystal structure	Appearance
Magnetite	Fe_3O_4	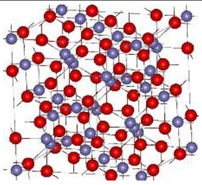<br>https://en.wikipedia.org/wiki/File:Magnetite_structure.jpg	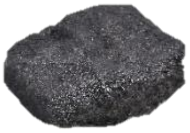<br>https://www.amazon.com/Raw-Magnetite-Mineral-Specimen-Classrooms/dp/B081BCMD82
Pyrite	FeS_2	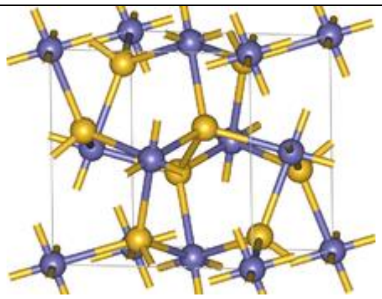<br>https://en.wikipedia.org/wiki/Pyrite	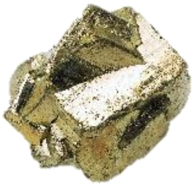<br>https://www.britannica.com/science/pyrite
Siderite	$FeCO_3$	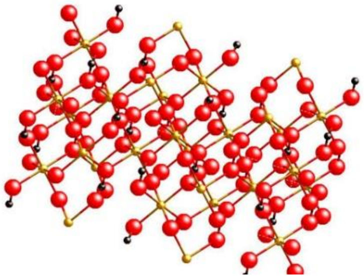<br>http://sci-culture.com/advancedpoll/GCSE/sedimentaryrocks.html	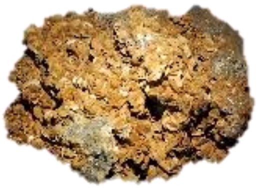<br>https://en.wikipedia.org/wiki/Siderite
Vivianite	$Fe_3(PO_4)_2{\cdot}8H_2O$	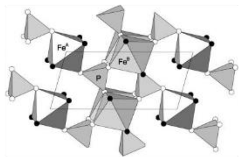<br>https://mineralcollectionblog.wordpress.com/2018/07/31/vivianite-group-minerals/	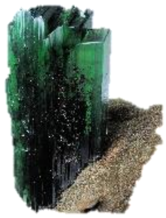<br>https://en.wikipedia.org/wiki/Vivianite
